# An elective two-week interdisciplinary acute rehabilitation program improves functional and speech outcomes in patients with Parkinson's disease

**DOI:** 10.3389/fresc.2025.1672759

**Published:** 2026-01-22

**Authors:** Steven Markos, Janice P. Dibling, Hayk Petrosyan, Stacey Chung, Katherine Toole, Sara J. Cuccurullo

**Affiliations:** 1 Department of Physical Medicine and Rehabilitation, Hackensack Meridian JFK Johnson Rehabilitation Institute, Edison, NJ, United States; 2 Department of Physical Medicine and Rehabilitation, Rutgers-Robert Wood Johnson Medical School, New Brunswick, NJ, United States; 3 Department of Physical Medicine and Rehabilitation, Hackensack Meridian School of Medicine, Nutley, NJ, United States

**Keywords:** functional improvement, inpatient rehabilitation, multidisciplinary rehabilitation, neurorehabilitation, Parkinson's disease

## Abstract

**Objective:**

The aim of this study was to examine the effects of an intensive, multidisciplinary, inpatient rehabilitation program on the functional outcomes of patients with Parkinson's disease (PD), admitted directly from home.

**Design:**

Retrospective cohort study.

**Setting:**

Suburban academic medical center with an inpatient rehabilitation facility.

**Methods:**

The study included 37 patients with PD who participated in a two-week acute inpatient multidisciplinary rehabilitation program. Functional assessments utilizing the Activity Measure for Post-Acute Care (AM-PAC), Section GG of the Inpatient Rehabilitation Facility–Patient Assessment Inventory (Section GG scores), and Voice Handicap Index (VHI) were collected and compared upon admission and discharge from the program.

**Results:**

Significant improvements were observed in the AM-PAC domains of Basic Mobility and Daily Activity between admission and discharge (*p* < 0.0001), as well as in Section GG scores for self-care and mobility (*p* < 0.001). Importantly, VHI scores demonstrated statistically significant improvements in vocal functioning (*p* = 0.028). No significant changes were observed in the Applied Cognitive domain of the AM-PAC (*p* = 0.871).

**Conclusions:**

Intensive inpatient rehabilitation programs have the potential to enhance both physical functioning and speech and vocal abilities in patients with PD. This study is the first of its kind in the United States, demonstrating the safety and efficacy of a multidisciplinary acute inpatient rehabilitation program designed to enhance the functional outcomes of individuals with PD living at home.

## Introduction

Parkinson's disease (PD) is a neurodegenerative disorder characterized by numerous motor and non-motor symptoms. PD is the fastest-growing neurologic disorder worldwide, with a projected global prevalence exceeding 12 million by 2040 (1). In the United States alone, the economic burden is expected to surpass $79 billion by 2037 (2). Motor symptoms of PD include rigidity, bradykinesia/akinesia, tremor, postural instability, and impairment of balance and gait. Other symptoms include autonomic dysfunction, cognitive symptoms, motor speech deficits, dysphagia, psychiatric disorders, depression, apathy, fatigue, and sleep disturbances (3–5). The sequelae of the motor and non-motor symptoms of PD can have significant lasting negative effects on quality of life (QOL), increase fall risk, impair mobility and functional independence, and affect social, emotional, and medical well-being.

Since there is currently no cure for PD, the objectives of treatments are to control and manage symptoms as well as slow disease progression. Main treatment options include pharmacological management, surgery, physical therapy, occupational therapy, and speech therapy. It has been postulated that exercise may have a neuroprotective and neurorestorative effect, achieved by modulating dopamine and glutamate neurotransmission, and increasing neurotrophic factor signaling to promote and enhance neuroplasticity (6). Previous studies have demonstrated significant improvements in motor symptoms following inpatient rehabilitation programs for patients with PD (7–11). Moreover, multidisciplinary clinical care teams are critical for the effective management of PD; recommendations have been established outlining the elements for optimal care, members of the team, role of patient and care partner, coordination of the team, team meetings, inpatient or outpatient care, and telehealth (12). Core members of the team should include the physiatrist, physical therapist, occupational therapist, speech-language pathologist, movement disorder neurologist, rehabilitation nurse, exercise physiologist, rehabilitation psychologist, social worker, dietician, and pharmacist (12).

Intensive rehabilitation programs involving multidisciplinary teams have demonstrated significant benefits for patients with PD (7–11). Results demonstrate that these programs improve motor function, including gait, balance, and dexterity, with improvements often sustained for several weeks or months (13, 14). Additionally, patients reported improvements in depression, quality of life, and functional independence. Some studies suggest that intensive rehabilitation may even slow the progression of motor symptoms and the need for medication adjustments (11, 12, 15–17).

Current research, however, offers limited evidence regarding the effects of interdisciplinary rehabilitation programs on vocal and speech functioning in individuals with PD. Patients with PD commonly experience hypokinetic dysarthria, which adversely affects all aspects of their speech (18). Studies report that approximately 90% of patients with PD exhibit communication disorders, with vocal issues being the most prominently affected and often arising early in the progression of the disease (19, 20). Individuals with PD often exhibit reduced variation in both pitch and loudness in their vocalizations; however, quantitative assessments of these vocal changes have not been thoroughly documented (18, 21). To develop a thorough understanding of these vocal characteristics within this population, it is imperative to include patient-centered measurements (22, 23). Ineffective communication abilities can profoundly affect the quality of life, leading to frustration for both the individuals affected and their caregivers. It is essential to recognize that many individuals with PD may not initially acknowledge their vocal impairments, resulting in self-assessments that underestimate the extent of their deficits. Additionally, there is a lack of research in the United States that examines the implementation and efficacy of multidisciplinary inpatient rehabilitation programs specifically designed for patients with PD. This may be largely attributed to constraints in insurance coverage. The majority of existing literature originates from studies conducted in various international settings, underscoring the pressing need for studies investigating the immediate and long-term benefits of these interventions, which could yield valuable insights into improving patient outcomes and the quality of life for patients living with PD in the United States.

The aim of this study is to examine the effects of a two-week intensive, multidisciplinary, inpatient rehabilitation program on motor and vocal functional outcomes in patients with PD. The Parkinson's Wellness Program (PWP) at the JFK-Johnson Rehabilitation Institute is a two-week acute inpatient rehabilitation program that admits patients living in the community for the multidisciplinary management of mobility, activities of daily living, speech, self-care deficits, as well as medical conditions.

## Methods

### General design

This is a retrospective analysis of prospectively collected data from patients who participated in a two-week acute inpatient multidisciplinary rehabilitation program between February 2022 and April 2024. This study included patients who were 1) at least 18 years old; 2) had a diagnosis of PD; 3) were admitted to an inpatient rehabilitation hospital directly from home; and 4) had completed the two-week inpatient rehabilitation program. Exclusion criteria entailed the patient's or decision-maker's dissent to enter the program. All patients who had pre- and post-assessments were included in the sample, resulting in a sample size of 37 participants. All clinical and demographic data were extracted from the electronic medical records, including patient comorbidities, the progression of the hospital stay, medications, and functional outcomes. The study was approved by the Institutional Review Board at Hackensack Meridian Health with a waiver of the informed consent form before the initiation of the study and data collection and was conducted in accordance with the Declaration of Helsinki. The Strengthening the Reporting of Observational Studies in Epidemiology Checklist (STROBE) was used to structure the manuscript and as a reporting guideline.

### Intervention

The PWP is a two-week inpatient rehabilitation program that encompasses a comprehensive array of multidisciplinary therapeutic interventions. Patients deemed appropriate candidates for the PWP included: those with a significant recent decline from prior level of function, those with a recent decline from prior level of function undergoing outpatient therapy programs but failing to meet goals, those with medical comorbidities requiring daily physician oversight to safely participate in physical or occupational therapy, and those with a chronic decline from prior level of function who were deemed unlikely to benefit from a less intensive outpatient therapy program. Criteria for discontinuing the program included the patient's or decision-maker's desire to leave, as well as intervention intolerance. Patients participating in the program received an average of three hours of skilled therapy per treatment day, which included physical therapy (1 h), occupational therapy (1 h), and speech therapy (1 h) each day for a total of 11 complete treatment days. Therapy was provided on an individualized basis, based on the patients’ needs, and the duration of each session was one hour. Key components of skilled therapy sessions included gait analysis and training, DME needs assessment and training, balance training, postural correction, amplitude training with emphasis on large-amplitude exercises, strengthening, and endurance training, for physical therapy based on the LSVT BIG program. Occupational therapy sessions included training for activities of daily living, fine-motor skill training, education on improved techniques, and home exercise programs incorporating the LSVT BIG program methodology. Speech therapy sessions included daily exercises that were focused on speaking with intent, encompassing increased volume, articulation, intelligibility, and projection based on SPEAK OUT! Program. During the sessions, sound level meters were utilized in order to provide quantifiable, visually presented volume measurements in decibels. In addition, cognition and swallowing were assessed for each participant and addressed based on individual needs. All physical and occupational therapists were Lee Silverman voice treatment (LSVT) BIG ® certified, while all speech-language pathologists were SPEAK OUT!® certified (23).

In addition to the therapeutic services, patients received daily oversight by the attending physiatrist, 24 h/day of skilled nursing care, and social worker support for discharge planning. As part of the admission process, a physical medicine and rehabilitation physician conducted a comprehensive evaluation to assess functional deficits and activity tolerance. For each patient, a neurologist with a specialization in movement disorders was consulted. Neurological assessments and medication alteration were conducted as deemed clinically necessary.

In addition to skilled therapy, the program provided enhanced interdisciplinary rehabilitation services to all patients and their families participating in the inpatient rehabilitation program. A rehabilitation psychological consultation was provided for all patients with follow-up as needed. Patients and caregivers received education from an inpatient pharmacist regarding medications, adequate understanding of the dosages, therapeutic effects, side effects, interactions, and any dietary considerations/interactions. Patients and caregivers received education from a clinical dietician to counsel the patient on timing of protein ingestion around medication scheduling to increase the maximum absorption benefit of the pharmaceutical regimen. Nursing-provided education was also delivered with a binder of multidisciplinary informational handouts. Patients were also offered 1 h of recreational therapy five times per week. Additionally, participants were exposed to a demonstration of available outpatient Rock Steady boxing, ParkinSINGS Choir programs, and educated about the need for follow-up care in order to maintain and enhance the skills achieved during their inpatient rehabilitation program.

### Outcome Measures

The functional assessments collected include the Activity Measure for Post-Acute Care (AM-PAC), Section GG of the Inpatient Rehabilitation Facility—Patient Assessment Inventory (Section GG scores), and the Voice Handicap Index (VHI) for evaluating vocal function. These assessments were administered at both the time of admission and discharge from the program. The AM-PAC assessments are designed to evaluate activity limitations across three distinct functional domains: Applied Cognitive Skills (AC), Daily Activity (DA), and Basic Mobility (BM). The AM-PAC exhibits outstanding test-retest reliability, with an intraclass correlation coefficient ranging from 0.91 to 0.97 (24). These domains are sensitive to changes in both positive and negative directions and have been validated across a diverse array of functional severities and diagnoses. Scores for the BM domain range from 11.95 to 104.9, the DA domain range from 2.73 to 115.4, and the AC domain range from 6.84 to 68.28 with higher scores indicating better functioning (25). AM-PAC assessments are conducted by certified therapists for all patients upon admission to the rehabilitation hospital and discharge.

In accordance with the Improving Post-Acute Care Transformation Act, all post-acute care settings are utilizing Section GG scores for the assessment of self-care and mobility. Section GG consists of 22 items that evaluate patients’ functional capabilities based on the type and extent of assistance required in the domains of mobility and self-care with higher scores indicating better functioning. The validity of Section GG scores has been established through a comprehensive study involving a substantial cohort of Medicare beneficiaries in inpatient rehabilitation settings (26). Section GG scores for mobility and self-care were collected at the time of both admission and discharge from the hospital.

Vocal functioning data were collected as speech-language pathologists administered the Voice Handicap Index (VHI) to each patient at both the time of admission and at discharge from the program. The VHI is a patient-reported outcome measure that has been extensively adopted and utilized in the PD population, demonstrating a significant responsiveness to interventions (27). The VHI is a widely recognized assessment tool that evaluates the impact of voice-related disabilities on the quality of life of patients, with higher scores indicating more severe deficits (28).

Functional assessments were performed on day 2 following the admission of the patient into the program to evaluate functional deficits and develop an appropriate treatment plan. Re-assessments were conducted on day 13, enabling a discussion regarding the patient's progress with the patient and/or family to prepare for discharge from the program.

### Data analysis

Statistical analyses were conducted using R software (version 4.0.3, 2020-10-10). Descriptive results for continuous variables were summarized using medians and interquartile ranges (IQRs), while categorical variables were reported as counts and percentages. Both univariable and multivariable methods were utilized to analyze data. The primary outcomes included changes in AM-PAC subscale scores for Basic Mobility (BM), Daily Activities (DA), and Applied Cognitive (AC), as well as Section GG scores for self-care and mobility and VHI scores. Changes in functional scores between admission and discharge were analyzed using exact Wilcoxon signed rank tests to account for non-normal distributions. Pearson's linear correlation coefficients were used to assess the relationships between changes in functional scores (AM-PAC, Section GG scores, and VHI) and various covariates, including age, chronicity of PD diagnosis, and the Levodopa equivalent dose. Multivariable analysis employing linear regression models was utilized to evaluate the simultaneous effects of confounding variables on the change in functional outcome measures between admission and discharge from the program. Each functional outcome measure was assessed separately with its own multivariable linear regression model. Results are reported as regression coefficients and 95% confidence intervals (95% CI). The significance level for all procedures was set at *p* < 0.05.

## Results

A total of 37 patients were included in the analysis. Table 1 demonstrates descriptive characteristics of patients admitted to the inpatient multidisciplinary rehabilitation program. Of the 37 patients, 27 were males (73%) with a median age of 74 years (IQR: 66–79). The median time since PD diagnosis for participants was 10 years (IQR: 7–16), with a median L-dopa equivalent dose of 585 mg/day (IQR: 300–900). The median baseline score on the Movement Disorder Society-Unified Parkinson's Disease Rating Scale (MDS-UPDRS) Part III was 34 (IQR: 27.5–42), based on data from 25 of the 37 patients. The median length of stay in the program was 13 days (IQR: 12–14).

**Table 1:** Demographic and clinical characteristics of study participants.

Characteristic	Value
Age (years), median (IQR)	74 (66–79)
Race, *n* (%)	
Black/African American	1 (8.1)
Asian	3 (2.7)
White	33 (89)
Gender, *n* (%)	
Male	10 (27)
Female	27 (73)
Disease duration (years), median (IQR)	10 (7–16)
MDS-UPDRS Part III, median (IQR), *n* = 25	34 (27.5–42)
Levodopa equivalent dose, median (IQR)	585 (300–900)
Length of stay, median (IQR)	13 (12–14)
Discharge disposition, *n* (%)	
Home	37 (100)

IQR, interquartile range; MDS-UPDRS, movement disorder society-sponsored unified parkinson's disease rating scale.

**Figure 1:**
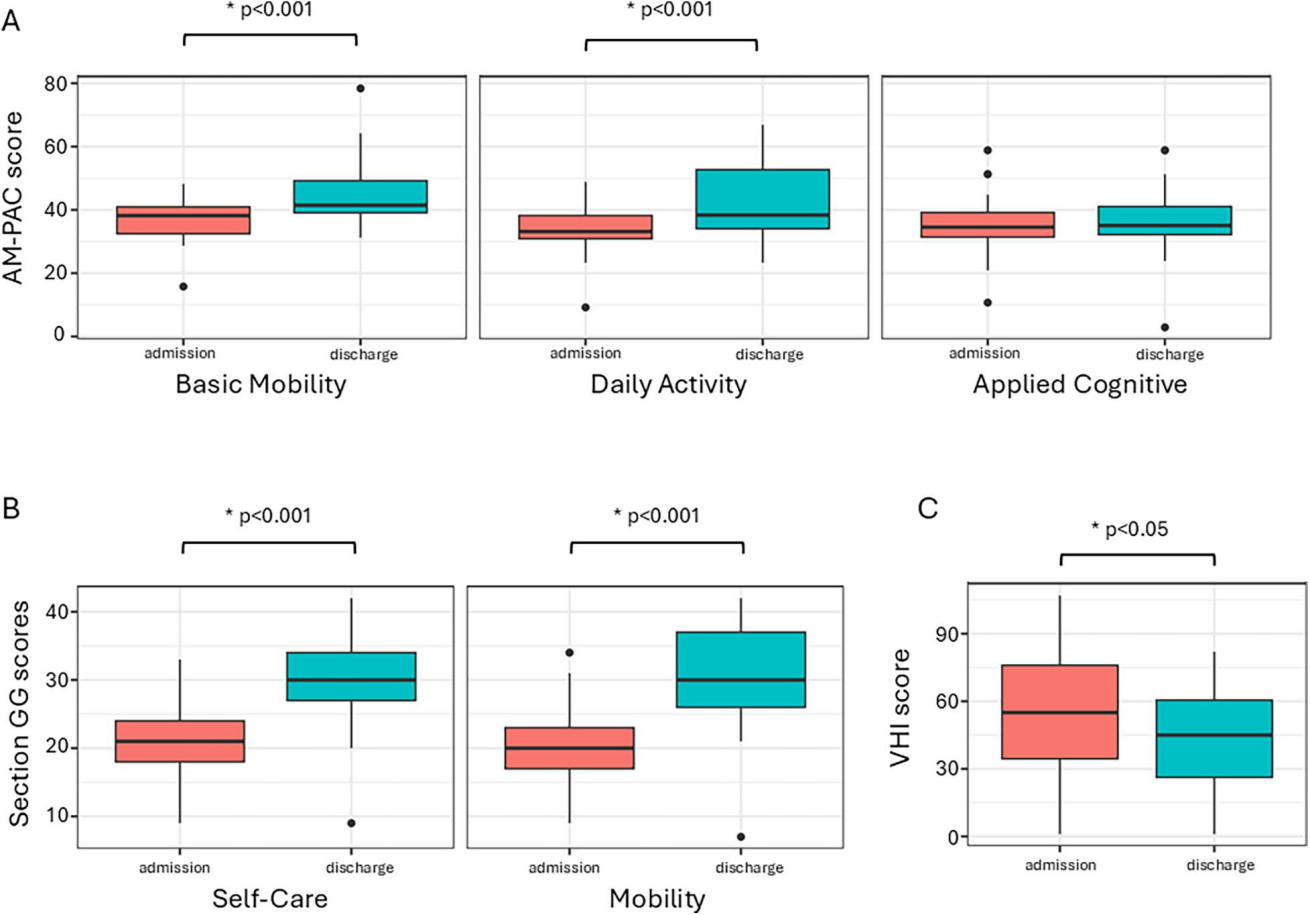
Boxplots depicting the changes in **(A)** AM-PAC scores across all three functional domains, **(B)** section GG scores, and **(C)** voice handicap Index (VHI) scores following participation in the program. The findings demonstrate statistically significant improvements in the basic mobility and daily activity domains of the AM-PAC, as well as in the self-care and mobility domains of the Section GG scores, and vocal functioning.

The results demonstrate that patients achieve significant gains in their functional abilities following participation in the program (Figure 1). Table 2 summarizes the functional assessment scores evaluated at admission and discharge from the program. Significant improvements were observed in AM-PAC scores for BM and DA between admission and discharge. Median BM scores increased from 38.38 (IQR: 8.71) to 41.48 (IQR: 9.70; *p* < 0.0001), and median DA scores improved from 33.16 (IQR: 7.37) to 38.13 (IQR: 14.55; *p* < 0.0001). However, no significant changes were observed in Applied Cognitive scores, with median values shifting minimally from 34.55 (IQR: 8.48) to 34.83 (IQR: 8.41; *p* = 0.8718) (Figure 1). Similarly, Section GG scores for self-care and mobility also showed significant improvements between admission and discharge from the program (Figure 1B). Median self-care scores increased from 21.00 (IQR: 6.00) to 30.00 (IQR: 7.00; *p* < 0.0001), and mobility scores rose from 20.00 (IQR: 6.00) to 30.00 (IQR: 11.00; *p* < 0.0001) (Figure 1B).

**Table 2:** Comparison of functional outcomes between admission and discharge from the program.

	Admission	Discharge	*p*-value
AM-PAC, *n* = 35	Mean (SD)	Mean (SD)	
Basic mobility	36.87 (5.98)	44.74 (9.84)	*p* < 0.001
Daily activities	33.67 (7.27)	42.03 (11.64)	*p* < 0.001
Applied cognitive	35.49 (9.16)	36.25 (10.05)	*p* = 0.871
Section GG scores, *n* = 37			
Mobility	20.59 (5.70)	30.97 (7.69)	*p* < 0.001
Self-care	20.86 (4.92)	30.43 (7.29)	*p* < 0.001
VHI scores, *n* = 34	53.12 (28.54)	43.41 (22.19)	*p* = 0.028

AM-PAC, activity measure for post-acute care; VHI, voice handicap index; SD, standard deviation.

*P* values indicate the results of pairwise Wilcoxon signed-rank tests.

Importantly, the evaluation of Voice Handicap Index (VHI) scores revealed significant improvements in vocal functioning, indicating enhanced quality of communication and expression (Figure 1C). Specifically, the median VHI scores decreased from 52.00 (IQR: 42.50) at the time of admission to 45.00 (IQR: 34.25) at discharge (*p* = 0.0289) (Figure 1C).

A series of correlation analyses, detailed in Supplementary Table S1, revealed a significant relationship between changes in participants’ Section GG scores for self-care and mobility (*r* = 0.70, *p* < 0.01) and between AM-PAC BM and DA scores (*r* = 0.78, *p* < 0.01). Positive correlations were also observed between changes in Section GG scores for self-care and AM-PAC BM scores (*r* = 0.41, *p* = 0.01) as well as between changes in Section GG scores for mobility and AM-PAC BM scores (*r* = 0.44, *p* = 0.01). However, changes in AM-PAC AC scores were not significantly correlated with other functional scores. Similarly, changes in participants’ VHI scores did not correlate with changes in other functional scores (see Supplementary Table S1). Additionally, there was no significant correlation between changes in participants’ functional scores and demographic or clinical variables such as age and disease duration or Levodopa equivalent dose (see Supplementary Table S1). These findings suggest that functional improvements in mobility, self-care, and vocal functioning were independent of baseline patient characteristics.

A linear regression analysis was performed to evaluate the impact of various confounding variables on functional recovery. The covariates included race, age, years since PD diagnosis, length of stay at the program, and the Levodopa equivalent dose as covariates. The results further confirmed the limited predictive value of demographic and clinical variables on functional gains, as presented in Table 3. No significant associations were observed between age, race, disease duration, Levodopa dose, or length of stay, and changes in AM-PAC BM, DA, or AC scores, Section GG scores for self-care or mobility, or VHI scores (*p* > 0.05). The regression coefficients and confidence intervals indicate that these variables did not have a statistically significant effect on the observed functional changes (Table 3).

**Table 3:** Multiple linear regression analysis models (each functional outcome was tested as a separate model) assessing the influence of demographic and clinical characteristic factors on functional changes, as measured by AM-PAC, section GG scores, and VHI scores.

Variable	AM-PAC: basic mobility		AM-PAC: daily activity		AM-PAC: applied cognitive		Section GG: self-care		Section GG: mobility		VHI	
	B (95% Cl)	*p*	B (95% Cl)	*p*	B (95% Cl)	*p*	B (95% Cl)	*p*	B (95% Cl)	*p*	B (95% Cl)	*p*
Race (Non-white/White).	−0.37 (−11 to 9.8).	0.94.	0.25 (−12 to 12).	0.97.	1.8 (−7.4 to 11).	0.69.	−1.2 (−8.5 to 6.1).	0.75.	−1.2 (−8.5 to 6.1).	0.75.	−0.10 (−30 to 30).	>0.99.
Age	−0.07 (−0.54 to 0.39).	0.75.	−0.14 (−0.68 to 040).	0.60.	−0.20 (−0.61 to 0.22).	0.34.	−0.03 (−0.034 to 0.28).	0.84.	−0.03 (−0.34 to 0.28).	0.84.	−0.18 (−1.3 to 0.99).	0.76.
Disease duration.	−0.26 (−0.67 to 0.16).	0.22.	0.01 (−0.48 to 0.50).	0.96.	0.03 (−0.34 to 0.41).	0.86.	−0.06 (−0.36 to 0.24).	0.68.	−0.06 (−0.36 to 0.24).	0.68.	−0.57 (−1.7 to 0.59).	0.32.
Levodopa equivalent dose.	0.00 (0.00 to 0.00).	0.96.	0.00 (0.00 to 0.01).	0.53.	0.00 (0.00 to 0.01).	0.12.	0.00 (0.00 to 0.00).	0.39.	0.00 (0.00 to 0.00).	0.39.	−0.01 (−0.02 to 0.00).	0.17.
Length of stay.	0.61 (−0.64 to 1.9).	0.33.	0.91 (−0.55 to 2.4).	0.21.	−0.48 (−1.6 to 0.64).	0.38.	0.25 (−0.65 to 1.1).	0.58.	0.25 (−0.65 to 1.1).	0.58.	0.15 (−3.8 to 4.1).	0.94.

AM-PAC, activity measure for post-acute care; VHI, voice handicap index; CI, confidence intervals.

No significant difference was observed for any of the confounding variables.

## Discussion

The findings of this study demonstrate that patients with PD living at home who participated in a 2-week multidisciplinary intensive inpatient rehabilitation program exhibited significant improvements in their functional outcomes. These improvements included enhanced mobility, improved ability to perform activities of daily living, and improved vocal functioning. Additionally, the results indicate that the inpatient rehabilitation program is safe, with no reported adverse events. To our knowledge, this study is the first of its kind in the United States, describing a program designed to admit patients with impairments from PD who live at home into a rehabilitation hospital to receive two weeks of multidisciplinary rehabilitation services under the supervising care of a physical medicine and rehabilitation physician, with the goal of improving both functional and medical status. Importantly, statistically significant improvements in function were reported by both patients and treating clinicians, as the AM-PAC and the VHI are patient-reported measures, and Section GG scores in mobility and self-care were determined by treating therapists.

Various outpatient programs have been established to support individuals with PD. However, only limited studies examined the impact of a multidisciplinary approach conducted in inpatient settings for patients who are living at home. Both observational and experimental studies have demonstrated the therapeutic effects and benefits of multidisciplinary inpatient rehabilitation programs for patients with PD (7–9, 15–17). While the duration and components of these studies vary, our results are in agreement with the existing literature, which indicates a positive impact and significant benefits for individuals living with PD. One retrospective observational study has demonstrated significant improvements in daily functional disability and a reduced risk of falls following a goal-oriented multidisciplinary rehabilitation program (16). This program consisted of a minimum of two rehabilitative sessions each day, conducted over 5–7 days per week, resulting in a total of at least 500 min of therapy per week (16). Our findings, reflected in the improved AM-PAC scores, indicate similar advancements, as the AM-PAC BM domain assesses physical function concerning ambulation, balance, and related daily activities. Notably, the study also revealed enhancements in cognitive abilities assessed through the Mini-Mental State Examination (16). However, our results did not indicate notable changes in the AC domain of the AM-PAC, which evaluates cognitive functioning in patients. One potential explanation for this observation is that the AM-PAC may lack sensitivity in detecting changes related to abstract reasoning, as reported in that study. Additionally, it is important to note that the study included cognitive therapy sessions as part of its multidisciplinary program (16). Furthermore, variations in cognitive outcomes may be influenced by the stage of the disease or the baseline severity of cognitive impairment. Future research is warranted to offer additional insights into the immediate effects of inpatient rehabilitation programs on the cognitive abilities of individuals with PD.

Several other studies have documented the positive impact of multidisciplinary inpatient programs, which range from two to six weeks in duration, on physical functioning. Participants in these programs have shown significant improvements across various aspects of motor function, which include mobility, ambulation, balance, and upper extremity function. Particularly, improvements have been observed using the Movement Disorders Society Unified Parkinson's Disease Rating Scale, the Timed Up-and-Go test, the Berg Balance Scale, the 10 m walk test, the 6 min walk distance, Functional Independence Measure (FIM), and the Short Physical Performance Battery (SPPB) (7–9, 15, 29). The improvements reported in these studies align with our findings, providing further evidence of the successful application of these improvements to the activities of daily living. This translates into a reduction in the functional limitations experienced by patients with PD in their daily routines, as demonstrated by observed improvements in AM-PAC BM and DA scores.

Additionally, several studies have reported that these programs provide significant benefits across multiple aspects of patients’ lives, including improvements in self-perceived quality of life and reduction in depressive symptoms (30, 31). This underscores the effectiveness of comprehensive approaches in improving the physical functioning and overall health of patients living with PD through inpatient multidisciplinary programs.

Another significant finding of this study is that our results indicate improvements in vocal functioning following participation in the inpatient rehabilitation program. Through active participation in the PWP program and dedicated engagement in intensive treatment, they benefit from listening to recordings of their own voices, utilizing sound level meters, and interacting with a diverse range of healthcare professionals. Consequently, while post-treatment scores on the VHI might indicate a decline in function, this change often signifies a more informed and accurate perspective regarding their communication challenges and overall effectiveness. As in the initial administration, some patients are simply unaware of how greatly their vocal quality has been reduced. Our results demonstrating improvements in VHI scores reflect meaningful improvements in vocal functioning as reported by patients. Improvements in intelligibility and volume have a significant impact on a person's quality of life. Improved VHI scores often translate into meaningful gains in real-life functioning, suggesting that an individual experiences their voice as less of a barrier in daily life. This can mean feeling more confident and less anxious in social situations, and finding it less physically taxing to speak.

Importantly, our results reveal that improvements in VHI scores do not correlate with improvements in the Section GG or AM-PAC scores. This observation underscores the unique and individual nature of symptom presentation and severity among patients. It is important to note that patients may experience deficits in motor function or vocal function at different stages of the disease and are independent of one another. Our results suggest that the multidisciplinary approach employed in our inpatient rehabilitation program significantly improves these functions in an independent manner. These findings indicate a strong correlation between AM-PAC and Section GG scores. Notably, we observed a significant association between improvements in AM-PAC BM scores and Section GG scores for mobility and self-care. This finding highlights the applicability of AM-PAC for patients with PD in inpatient settings, as it has been previously demonstrated to reliably identify improvements during rehabilitation for patients with various neurological conditions, including stroke and musculoskeletal disorders (25, 32).

This study also investigated the potential influence of various predictors on improvements within the rehabilitation program. We analyzed whether factors such as age, race, Levodopa equivalent dose, the chronicity of PD diagnosis, and the length of stay in the program can impact the outcomes. The MDS-UPDRS score was excluded from this analysis due to incomplete data. Our findings indicate that none of these factors significantly impacted the program's results. These outcomes demonstrate the effectiveness of a multidisciplinary inpatient rehabilitation program for patients with PD, independent of their demographic or medical characteristics. This is particularly noteworthy given that the duration of the disease and Levodopa equivalent dose may reflect the severity of the condition, as our patient cohort included individuals with disease durations ranging from 7 to 16 years, and the median score on the MDS-UPDRS Part III was 34, spanning 14–48. It is essential to highlight that the program demonstrates effectiveness across a spectrum of patient profiles.

While it stands to reason that the benefits of this program may reduce the risk of morbidity, mortality, emergency room visits, or hospitalization, this potential effect has not yet been quantified. Future studies with wide-ranging outcome measures may produce additional metrics that could improve through this type of rehabilitation program. Additionally, a more robust set of data and a larger sample size demonstrating the positive impact of this type of rehabilitation program may lead to a higher likelihood of insurance authorizations allowing patients to undergo this program and receive its benefits. The limitations of this study include the absence of a control group for comparison and the lack of blinding for both patients and healthcare providers during assessments. Additionally, the absence of a disease stage presents a limitation, as it could enhance the analysis and enable effective comparison with the results of other relevant studies. Furthermore, the lack of long-term follow-up data to demonstrate the sustainability of the program's benefits represents an essential aspect for consideration in future research initiatives. Another important direction for future research will be the financial impact of a program like this. It remains to be determined whether a proactive intervention, although associated with an immediate cost for these services, can reduce overall costs by preventing future morbidity and associated higher costs.

Overall, this study suggests that an interdisciplinary rehabilitation program can provide clinically significant benefits to patients living in the community with PD. These benefits can extend to caregivers as well by increasing patient independence and decreasing caregiver burden, and improving patients’ overall quality of life as evidenced by significant improvements in patient-reported scores.

## Data Availability

The raw data supporting the conclusions of this article will be made available by the authors, without undue reservation.
