# Supplementary material for: An elective two-week interdisciplinary acute rehabilitation program improves functional and speech outcomes in patients with Parkinson's disease
Source: Front Rehabil Sci. 2026 Jan 22;6:1672759. doi: 10.3389/fresc.2025.1672759 (PMC12872888; doi:10.3389/fresc.2025.1672759)
Supplement: Supplementary file 1 [file Table1.docx]

Supplemental Table 1. An examination of the correlation between the demographic and medical characteristics of participants and the functional changes observed following their participation in the program.

| **Variable** | **Variable** | **r** | **p-value** |
| --- | --- | --- | --- |
| Section GG: Self-Care | Section GG: Mobility | 0.70 | **0.00** |
| Section GG: Self-Care | AM-PAC Basic Mobility | 0.41 | **0.01** |
| Section GG: Mobility | AM-PAC Basic Mobility | 0.44 | **0.01** |
| Section GG: Self-Care | AM-PAC Daily Activity | 0.32 | 0.06 |
| Section GG: Mobility | AM-PAC Daily Activity | 0.36 | **0.03** |
| AM-PAC Basic Mobility | AM-PAC Daily Activity | 0.78 | **0.00** |
| Section GG: Self-Care | AM-PAC Applied Cognitive | 0.26 | 0.13 |
| Section GG: Mobility | AM-PAC Applied Cognitive | 0.25 | 0.15 |
| AM-PAC Basic Mobility | AM-PAC Applied Cognitive | 0.22 | 0.20 |
| AM-PAC Daily Activity | AM-PAC Applied Cognitive | 0.23 | 0.18 |
| Section GG: Self-Care | VHI Score | 0.02 | 0.92 |
| Section GG: Mobility | VHI Score | 0.07 | 0.69 |
| AM-PAC Basic Mobility | VHI Score | 0.09 | 0.62 |
| AM-PAC Daily Activity | VHI Score | 0.11 | 0.55 |
| AM-PAC Applied Cognitive | VHI Score | -0.18 | 0.32 |
| Section GG: Self-Care | Age at Admission | 0.00 | 0.98 |
| Section GG: Mobility | Age at Admission | -0.15 | 0.37 |
| AM-PAC Basic Mobility | Age at Admission | 0.01 | 0.95 |
| AM-PAC Daily Activity | Age at Admission | -0.05 | 0.79 |
| AM-PAC Applied Cognitive | Age at Admission | -0.18 | 0.29 |
| VHI Score | Age at Admission | -0.04 | 0.83 |
| Section GG: Self-Care | Disease duration | -0.02 | 0.89 |
| Section GG: Mobility | Disease duration | -0.21 | 0.21 |
| AM-PAC Basic Mobility | Disease duration | -0.20 | 0.24 |
| AM-PAC Daily Activity | Disease duration | 0.09 | 0.60 |
| AM-PAC Applied Cognitive | Disease duration | 0.12 | 0.49 |
| VHI Score | Disease duration | -0.26 | 0.14 |
| Age at Admission | Disease duration | -0.19 | 0.25 |
| Section GG: Self-Care | Levodopa equivalent dose | 0.15 | 0.39 |
| Section GG: Mobility | Levodopa equivalent dose | 0.14 | 0.41 |
| AM-PAC Basic Mobility | Levodopa equivalent dose | -0.03 | 0.86 |
| AM-PAC Daily Activity | Levodopa equivalent dose | 0.15 | 0.39 |
| AM-PAC Applied Cognitive | Levodopa equivalent dose | 0.27 | 0.12 |
| VHI Score | Levodopa equivalent dose | -0.32 | 0.07 |
| Age at Admission | Levodopa equivalent dose | 0.03 | 0.86 |
| Disease duration | Levodopa equivalent dose | 0.26 | 0.11 |

r - Pearson’s linear correlation coefficient; AM-PAC – activity measure for post-acute care; VHI – voice handicap index. Bolded numbers indicate statistical significance.
